# Fluid Shear Stress Sensing by the Endothelial Layer

**DOI:** 10.3389/fphys.2020.00861

**Published:** 2020-07-24

**Authors:** Etienne Roux, Pauline Bougaran, Pascale Dufourcq, Thierry Couffinhal

**Affiliations:** ^1^Inserm, UMR 1034, Biology of Cardiovascular Diseases, University of Bordeaux, Bordeaux, France; ^2^UMR 8560 IHPST — Institut d’Histoire et de Philosophie des Sciences et des Techniques, CNRS, Université Paris 1 Panthéon-Sorbonne, Paris, France

**Keywords:** endothelial cell, shear stress, vasoreactivity, vascular remodeling, angiogenesis, regulation – physiological, tensegrity

## Abstract

Blood flow produces mechanical frictional forces, parallel to the blood flow exerted on the endothelial wall of the vessel, the so-called wall shear stress (WSS). WSS sensing is associated with several vascular pathologies, but it is first a physiological phenomenon. Endothelial cell sensitivity to WSS is involved in several developmental and physiological vascular processes such as angiogenesis and vascular morphogenesis, vascular remodeling, and vascular tone. Local conditions of blood flow determine the characteristics of WSS, i.e., intensity, direction, pulsatility, sensed by the endothelial cells that, through their effect of the vascular network, impact WSS. All these processes generate a local-global retroactive loop that determines the ability of the vascular system to ensure the perfusion of the tissues. In order to account for the physiological role of WSS, the so-called shear stress set point theory has been proposed, according to which WSS sensing acts locally on vessel remodeling so that WSS is maintained close to a set point value, with local and distant effects of vascular blood flow. The aim of this article is (1) to review the existing literature on WSS sensing involvement on the behavior of endothelial cells and its short-term (vasoreactivity) and long-term (vascular morphogenesis and remodeling) effects on vascular functioning in physiological condition; (2) to present the various hypotheses about WSS sensors and analyze the conceptual background of these representations, in particular the concept of tensional prestress or biotensegrity; and (3) to analyze the relevance, explanatory value, and limitations of the WSS set point theory, that should be viewed as dynamical, and not algorithmic, processes, acting in a self-organized way. We conclude that this dynamic set point theory and the biotensegrity concept provide a relevant explanatory framework to analyze the physiological mechanisms of WSS sensing and their possible shift toward pathological situations.

## Introduction

Blood flow through the vascular network produces mechanical forces exerted within the blood vessels, mainly the blood pressure, and the wall shear stress (WSS) exerted on the endothelial layer that lines the lumen of the vessel. Both forces are sensed by the cardiovascular system, which in turn modifies its activity and hence the mechanical characteristics of the blood flow. As a consequence, a dynamic causal loop exists between the morphofunctional state of the cardiovascular system, which mechanically determines the blood flow and the mechanical constraints it exerts on this system, and the blood flow which, through the mechanical sensing pathways stimulated by these constraints, modifies this morphofunctional state. Since the cardiovascular system develops early during embryonic development, being functional approximately around 10 weeks of gestation, mechanosensitivity may play an important role during prenatal and postnatal vascular morphogenesis as well as during adulthood. Recent studies have shown that the endothelial cell (EC) sensitivity to WSS is involved in several developmental and physiological vascular processes such as angiogenesis and vascular morphogenesis, vascular remodeling, and vascular tone (Chen and Tzima, 2009; Franco et al., 2015; Carter et al., 2016; Poduri et al., 2017; John et al., 2018; Iring et al., 2019). In contrast with barosensitivity, which involves central control by the brainstem of the heart activity and the peripheral arterial resistance and endocrine blood volume control, shear stress sensitivity seems to be determined and act locally. Despite the absence of central control, FSS sensitivity contributes to ensure an adequate behavior of the cardiovascular system as a whole, i.e., able to ensure adequate oxygen and nutrient delivery to the tissues. Regarding WSS sensitivity, the vascular system can hence be viewed as a self-organized homeostatic system. In order to account for the WSS-sensitive regulation of the vascular system, some authors have applied the general set point theory (SPT) of homeostatic processes to WSS-dependent vessel modeling (Baeyens et al., 2015; Baeyens and Schwartz, 2016). However, on the one hand, WSS sensitivity is involved in a variety of short-term and long-term modulations of the vascular activity, and, on the other hand, the classical SPT is initially a theory of central control. The question then arises whether this theory is applicable to the variety of behavioral processes determined by WSS. This is an important question since WSS sensing is a physiological phenomenon but is associated with several vascular diseases (Ong et al., 2020). If valid, the SPT would be a useful tool to understand how WSS sensing can shift from normal, beneficial processes toward pathological ones. The aim of this article is (1) to summarize the physical characteristics of the WSS applied to the vascular wall in physiological conditions, (2) to review the existing literature on the effects of WSS sensing on the behavior of ECs and its short-term (vasoreactivity) and long-term (vascular morphogenesis and remodeling) effects on vascular functioning in physiological condition, and (3) to analyze the relevance, explanatory value, and limitations of the SPT applied to WSS.

## Physical Characteristics of Fluid Shear Stress in Blood Vessels

### Theoretical Principles

**FIGURE 1:**
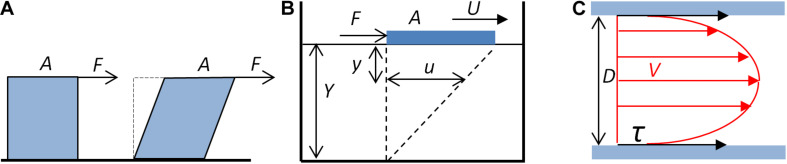
Fluid shear stress. **(A)** Schematic representation of the shear stress. The fluid exerts a tangential force *F* on the surface *A* of a solid (blue cube), which tends to deform the solid. **(B)** Shear stress, shear rate, and viscosity. A plate (blue block) of area *A* submitted to a tangential force *F* moves on the surface of a liquid of depth *Y*. The displacement at the surface of the liquid (*U*) generates, at the *y* distance to the surface, a movement of the fluid *u*. The shear rate is the ratio *du*/*dy*. For a Newtonian fluid, *du*/*dy* is constant. **(C)** Schematic representation of the shear stress exerted by the blood flow on the wall of a cylindrical vessel. Red arrows represent the velocity (*V*) of laminar blood flow. The shear stress (*τ*, black arrows) exerted by the blood on the wall of the tube depends on the blood flow mean velocity, the blood viscosity, and the diameter (D) of the tube (Eqs. 3 and 4).

The shear stress exerted on the vessel wall is a physical phenomenon that is the consequence of the frictional forces generated by the blood flow on the luminal surface of the wall. Unlike the pressure pulse, which creates a radial and circumferal strain that generates the distension of the artery wall, the frictional forces of the blood create a WSS tangential to the direction of the blood flow and the vessel axis. The WSS corresponds to the viscous drag that the fluid exerts, due to its viscosity, on the wall of the vessel, i.e., the lumen side of the endothelium. For an ideal fluid, for which the viscosity is null, there is no shear stress. The shear stress is schematically represented in Figure 1A.

The shear stress τ is then defined as the ratio of the tangential force *F* to the surface area *A* to which it is applied, and is hence a pressure.

(1) $$\tau =\frac{\text{F}}{\text{A}}$$

The shear stress depends on the flow and the rheological properties of the fluid, and the geometrical characteristics of the pipe through which it flows. Assuming several simplifications, it is possible to calculate the WSS exerted by the blood flow in a vessel segment, under the application of fluid mechanics principles (Marchandise et al., 2007; Fung, 2010; Caro, 2012; Koeppen et al., 2018). For a Newtonian fluid, for which the viscosity is constant, the shear stress depends on the viscosity of the fluid and the shear rate. This can be illustrated by a plate moving at constant velocity on a homogenous fluid, as shown in Figure 1B.

For a Newtonian fluid, the shear rate, defined as the ratio of the displacement τ of the fluid at a given distance *y*, d*u*/d*y*, is constant, and the viscosity η is the ratio of the shear stress to the shear rate d*u*/d*y*.

(2) $$\eta =\frac{\tau }{\text{d}\,u/\text{d}\,y}$$

Considering a vessel segment as a linear tube of constant diameter, and assuming the linear flow of a Newtonian fluid, application of the Poiseuille’s law allows formulation of the quantitative relationship between the shear rate as a function of the geometrical characteristic of the vessel segment and of the flow (Figure 1C). From the definition of the Newtonian viscosity, the shear stress is given by the product of the viscosity and the shear rate (Eq. 2). For a cylindrical segment, the shear rate α can be expressed as a function of the diameter *D* of the segment and the mean velocity *V*_*m*_.

(3) $$\alpha =\frac{8\times {\text{V}}_{m}}{\text{D}}$$

The shear stress can then be expressed as a function of the viscosity of the fluid, the mean flow velocity, and the diameter of the segment:

(4) $$\tau =\eta \times \frac{8\times {\text{V}}_{\text{m}}}{\text{D}}$$

For a cylindrical tube, the flow rate *Q* is the product of the mean velocity of the fluid and the cross sectional area of the tube. Hence, the mean velocity can be expressed as follows:

(5) $$V_{m}=\frac{4\times Q}{\pi \,D^{2}}$$

From Eqs 4 and 5, the shear stress exerted on a wall is proportional to the viscosity of the fluid and the flow rate and inversely proportional to the diameter *D* (or the radius *r*) of the vessel segment at the power 3:

(6) $$\tau =\eta \times \frac{32\,\text{Q}}{\pi \,{\text{D}}^{3}}=\eta \times \frac{4\,\text{Q}}{\pi \,{\text{r}}^{3}}$$

The shear stress can also be expressed as a function of the pressure differential in the segment, taking into account the Poiseuille’s equation:

(7) $$Q=\frac{\pi \,r^{4}}{8\,\eta \,L}\times \Delta \,P$$

*L* is the length of the segment and Δ*P* the pressure differential between the extremities of the segment. Replacing *Q* in Eq. 6 by Eq. 7 gives the following equation:

(8) $$\tau =\frac{\text{r}}{2}\times \frac{\Delta \,\text{P}}{\text{L}}$$

Under the simplifications already mentioned, the shear stress exerted on the vessel wall (WSS) can hence be calculated from the geometry of the vessel segment and the characteristics of the flow, either the mean flow velocity (Eq. 4) or the flow rate (Eq. 6), or, alternatively, the pressure differential in the segment (Eq. 8) (Reneman et al., 2006). The equations used to calculate experimental WSS values depend on the nature of the data obtained from the experiments to characterize the blood flow. Since measurement of the pressure differential is technically difficult, Eq. 8 is rarely used. Instead, Eqs 4 and 6 are usually applied to calculate the WSS in vessels. In experimental studies in which the flow rate is known, in particular in *in vitro* experiments, Eq. 6 is used, whereas in clinical studies in which the flow velocity is measured (allowing the calculation of the shear rate), Eq. 4 is used. When maximal velocity *V*_*max*_ instead of mean velocity is measured, Eq. 6 should be corrected in order to take into account the difference between *V*_*m*_ and *V*_*max*_. Due to the frictional force, the velocity is higher at the center of the segment, and, for a Newtonian fluid, the velocity profile is parabolic, and the ratio of *V*_*max*_ to *V*_*m*_ is 2. Hence,

(9) $$\tau =\eta \times \frac{4\times {\text{V}}_{\text{m}\,\text{a}\,\text{x}}}{\text{D}}$$

However, in *in vivo* studies, precise calculation of shear stress values should take into account the fact that the blood in a non-Newtonian fluid, namely, its viscosity, is not constant. An apparent viscosity can be attributed, i.e., the viscosity of a Newtonian fluid showing the same relationship between *Q* and Δ*P*, according to the Poiseuille’s law. Additionally, the velocity profile is flattened, which changes the ratio of *V*_*max*_ to *V*_*m*_(*V*_*max*_/*V*_*m*_). In arterioles, this ratio is usually between 1.39 to 1.54, i.e., less than the theoretical value 2 (Reneman et al., 2006). Also, for small vessels with diameters less than 300 μm, blood viscosity is lower than in larger vessels, so that shear stress may differ, due to change in apparent viscosity, depending of the location throughout the vascular tree.

### Shear Stress Patterns and Physiological Values

Additionally, these physical characterizations of FSS are done under simplifications, namely, considering a uniform and laminar blood flow through a straight cylindrical vessel segment, conditions that are not always realized in the vasculature. First, in arteries, blood flow is not constant but pulsatile, a consequence of the pulsatile activity of the heart. Second, the vessel segments are not always rectilinear, so that blood velocity and, hence, FSS is not similar at the inner and outer parts of the curved segment. Third, blood flow is not always linear. It can be turbulent, or disturbed, in particular at arterial bifurcations and curvatures. Numerous models based on computational fluid dynamics have been developed to predict these local patterns of blood flow and WSS, in particular for human aorta. Indeed, several pathologies, like plaque formation and atherosclerosis, are associated with zones of disturbed blood flow and low WSS, and such models are helpful tools to predict the progression of the disease and to choose the most appropriate treatment and its timing (Ong et al., 2020).

Despite these local complex patterns of WSS, average values have been calculated by several authors and are available in the literature, either in the international (SI) or the centimeter-gram-second (CGS) system of units (Lipowsky et al., 1978; Kamiya and Togawa, 1980; Koller and Kaley, 1991; Joannides et al., 1995; Cheng et al., 2003; Carter et al., 2016; Saw et al., 2017). In order to help comparing the values published in the literature and applying the equations in a coherent unit system, Table 1 gives a summary of units in both systems and their conversion ratio. Table 2 presents several *in vivo* measurements of WSS in large and medium arteries, arterioles, veins, and venules (Lipowsky et al., 1978; Cheng et al., 2003; Reneman et al., 2006). Other data are also given in Table 3.

**TABLE 1:** Comparative units.

Physical quantity	SI unit (symbol)	CGS unit
Force	Newton (N)	dyne = 1.10^–5^ N
Pressure	Pascal (Pa)	barye = dyne cm^–2^ = 1.10^–1^ Pa
Viscosity	pascal second (Pa s)	poise = barye second = 1.10^–1^ Pa s
Length	meter (m)	centimeter = 1.10^–2^ m
Velocity	meter/second (m s^–1^)	centimeter/second = 1.10^–2^ m s^–1^
Volume	meter^3^ (m^3^)	centimeter^3^ = 1.10^–6^ m^3^
Flow rate	meter^3^/second (m^3^ s^–1^)	centimeter^3^/second = 1.10^–6^ m^3^ s^–1^
Shear rate	second^–1^ (s^–1^)	second^–1^ (s^–1^)

*Units of basic physical quantities in the international system (SI) of units and the centimeter-gram-second (CGS) system of units.*

**TABLE 2:** *In vivo* WSS values in different vascular segments.

Vessel	hAA	hCCA	hBA	hCFA	hIVC	cMA	cMV
D (mm)	16.3	5.5–7.7	3.1–4.4	7.0	18.9	0.029	0.031
mWSS (Pa)	0.35	1.1–1.4	0.3–0.5	0.3–0.4	0.34	4.71	2.9
Viscosity (mPa S^–1^)	4	2.9–4.6	4.8–5.0		4	3.59	5.15

*hAA, human abdominal aorta; hIVC, human inferior vena cava (Cheng et al., 2003). hCC, human carotid artery; hBA, human brachial artery; hCFA, human common femoral artery (Reneman et al., 2006). cMA, cat mesenteric arteriole; cMV, cat mesenteric venule (Lipowsky et al., 1978). D, vessel diameter; mWSS, mean wall shear stress.*

**TABLE 3:** Change in blood flow rate and WSS-induced vasoreactivity.

	hCCA		hBA		rCrA	
			
	Initial	Final	Initial	Final	Initial	Final
flow rate (mL s^–1^)	*4.82*	*8.3*	0.4	1.22	*2.67* × *10*^–^*^6^*	*2.78* × *10*^–^*^6^*
diameter (cm)	0.639	0.675	0.267	0.277	2.17 × 10^–3^	9.78 × 10^–3^
WSR (s^–1^)	188	275	*214*	*583*	2,658	3,879
WSS (dyn cm^–2^)	*7*	*10*	*7*	*20*	*53*	*78*
Viscosity (Poise)	*0.035*	*0.035*	*0.035*	*0.035*	*0.02*	*0.02*
*S*_*WSS*_ (dyn cm^–1^)	5.91 × 10^–3^		3.85 × 10^–4^		1.60 × 10^–5^	

*Experimental data obtained in human common coronary artery (hCCA) (Carter et al., 2016), human brachial artery (hBA) (Joannides et al., 1995), and rat cremaster muscle arterioles (rCrA) (Koller and Kaley, 1991). WSR, wall shear rate; WSS, wall shear stress. Data in italics are calculated from the experimental data given by the authors using Eq. 7. The sensitivity coefficient *S*_*WSS*_ is defined as the ratio of change in diameter on change in WSS between initial and final conditions.*

It is classically considered that WSS in arteries is around 1–2 Pa (10–20 dynes cm^–2^), and around 0.1–0.6 Pa in veins (1–6 dynes cm^–2^), around 10-fold less (Ballermann et al., 1998; Baeyens et al., 2016). Compared to other mechanical forces to which the vessels are submitted, shear stress is very low. For example, mean arterial blood pressure is around 13 kPa, endogenous stress in tissue, as well as focal adhesion (FA) stress, is around 3–5 kPa (Galbraith and Sheetz, 1997; Balaban et al., 2001). Additionally, these average values should be taken as a range of order, since the values depend on where they have been measured and the mode of calculation. In humans, the mean WSS in the common carotid artery (CCA) is around 1.2 to 1.4 Pa, and the peak WSS around 2.5 to 3.6 Pa, whereas in common femoral, superficial femoral, and brachial arteries, the mean WSS ranges from 0.3 to 0.5 Pa (Reneman et al., 2006). The physiological average value of WSS is hence not uniform throughout the arterial network, but may vary locally.

Hence, WSS should be viewed as a physical constraint exerted on the endothelium of the vessels, characterized by a complex spatial and temporal pattern including its intensity, direction, pulsatility, and regularity. As already seen, WSS is about 1,000-fold less than other mechanical stresses, which means that WSS sensitivity is in a range quite different than other kinds of mechanical sensitivity. ECs are sensitive to small variations in magnitude, but also in the direction and regularity of blood flow-induced WSS (Givens and Tzima, 2016). These WSS characteristics are sensed and interpreted locally by the ECs, and this local WSS sensitivity has global physiological consequences. It is indeed an important determinant of the morphology of the vasculature, controlling both its development during embryogenesis and its remodeling during postnatal and adult life. It also modulates the vascular reactivity in response to short-term blood flow change. WSS sensitivity is hence an important physiological property that optimizes blood flow to the tissues and ensures the mechanical integrity of the vessel walls.

## Shear Stress and Vessel Physiology

Among the large variety of processes involving the endothelial sensitivity to WSS, the present analysis will focus on three main physiological responses to WSS, short-term vasoreactivity, long-term vessel remodeling, and vessel architecture building during vascular morphogenesis.

### Shear Stress and Vasoreactivity

Several studies have in past decades evidenced that shear-stress induces vasodilatation in an epithelium-dependent manner (Holtz et al., 1983; Vanhoutte, 1986). Inversely, reduction in blood flow has been shown to induce vasoconstriction, which mechanically increases WSS (Langille et al., 1989). *In vivo* experiments have shown that increase in WSS, either by increase in blood flow and wall shear rate (WSR) (Koller and Kaley, 1991; Carter et al., 2016), or fluid viscosity (Melkumyants and Balashov, 1990), triggered an arterial vasodilatation that occurred within a few seconds. This rapid increase in vessel diameter is due to the relaxation of the arterial musculature induced by WSS-induced endothelial release of vasorelaxant agonists, such as prostacyclin and, more importantly, nitric oxide (NO) (Frangos et al., 1985; Joannides et al., 1995). WSS-induced NO production is due to the phosphorylation of the NO synthase (NOS) at various serine sites, but the molecular interactions that couple WSS sensing and NOS phosphorylations are complex. Recent studies have shown that the mechanically gated ion channel PIEZO-1, located at the plasma membrane, and the mechanocomplex PECAM-1, vascular endothelial cadherin (VE-cadherin), and VEGFR2 complex, located at cell–cell junctions, are key molecular interactors in WSS sensitivity, but the ways they interplay are controversial. In the model proposed by Iring et al. (2019) PIEZO-1 activation by WSS induced autocrine or paracrine secretion of adrenomedulin and ATP. Each mediator induces NOS phosphorylation by specific intracellular pathways. Adrenomedulin binds to its membrane receptor CALCRL, which is coupled to G_s_ heterotrimeric G protein, triggering cAMP production, PKA activation and NOS phosphorylation. ATP binds to P2Y receptors coupled to G_q/11_ protein, inducing PLCβ activation, phosphorylation of the mechanocomplex, activation of PI3K, than of AKT, leading to NOS phosphorylation (Iring et al., 2019). However, it has been shown that PIEZO-1 is activated downstream, and not upstream, G_q/11_ protein (dela Paz and Frangos, 2019). Previous studies based on endothelial microtubules disruption have shown that the integrity of the cytoskeleton is necessary for WSS-induced vasodilatation (Sun et al., 2001), and suggested the existence of complex interplays between PIEZO 1 and EC cytoskeleton (Nourse and Pathak, 2017). Whatever the mechanisms responsible for WSS-induced vasoreactivity, its physiological consequence is a retroactive limitation of WSS variation. Indeed, for a given viscosity and flow rate, WSS is inversely proportional to the diameter of the vessel at the power 3 (see Eq. 6), so that physiological WSS-induced vasodilatation or constriction causes mechanically a variation in WSS opposite to its initial change.

### Shear Stress and Vascular Remodeling

In addition to short-term vasoactive response to WSS, sustained increase in WSS induces long-term remodeling of vessel walls. It has been shown, using arterovenous fistula techniques in dogs and monkeys to modulate the blood flow rate, which sustained increase in WSS-induced outward remodeling and subsequent growth in vessel caliber (Kamiya and Togawa, 1980; Zarins et al., 1987). As a mechanical consequence of the remodeling and increase in vessel diameter (see Eq. 7), the WSS that had initially increased returned to its initial value around six months post operatively. Inversely, decrease in blood flow induced by ligation has been shown to induce endothelium-dependent inward remodeling and constitutive reduction in artery diameter (Langille and O’Donnell, 1986; Langille et al., 1989). WSS-induced remodeling involves several processes affecting vessel homeostasis such as EC morphology, endothelial permeability, inflammation, etc. In adults, ECs lining the arterial vessel adopt differential morphologies according to the direction and the magnitude of the SS, and their alignment reflects the direction of the flow. Physiological laminar WSS promotes cell elongation and orientation in the direction of flow, suppresses proliferation, stimulates anti-inflammatory gene expression, and suppresses expression of inflammatory pathways. WSS below or above its physiological value induces changes in EC alignment, polarization, and gene expression and activates inflammatory response and remodeling processes (Zhou et al., 2014; Baeyens et al., 2016). The molecular interactors of these processes are multiple and related by complex and not fully understood interplays, but a key element of this WSS sensitivity is the mechanocomplex PECAM-1, VE-cadherin, and VEGFR2 (and possibly VEGFR3). Different reports have demonstrated that PECAM-1 acts in concert with VE-cadherin and VEGFR2 to mediate a large number of shear stress responses. These responses include EC alignment, NF-κB activation, and Akt phosphorylation following application of shear stress (Fleming et al., 2005; Tzima et al., 2005). Importantly, PECAM-1 is required for both anti-inflammatory and inflammatory signaling in ECs (Tzima et al., 2005). *PECAM-1* KO mice subjected to partial carotid artery ligation display defects in flow-mediated vascular remodeling and intima-media thickening, due to defects in the NF-κB pathway (Chen and Tzima, 2009). In the current model proposed by Baeyens et al. (2016), the PECAM-1, VE-cadherin, VEGFR complex, associated with the cytoskeleton, is the mechanotransducer that converts the mechanical stimulus into a biochemical signal responsible for the EC behavioral responses and the shift from the quiescent and steady state of the vessel to its inflammatory and remodeling one. Inward and outward remodeling in response to reduction and increase in WSS, respectively, tend to bring WSS back to its original value, at which the vessel returns to a quiescent state. In this process, VEGF receptors seem to determine the WSS value for this quiescent state, since changing expression of VEGFRs shifts the quiescent state WSS value (Baeyens et al., 2015).

### Shear Stress and Vascular Morphogenesis

Wall shear stress sensitivity is not only involved in vessel diameter adjustment in adults, but also contributes to the pattern of the vascular architecture during developmental vessel formation. A primary vascular plexus initially expands by sprouting (Isogai et al., 2003; Potente et al., 2011) followed by remodeling of vessel organization, shape, and size. Superfluous and inefficient connections are pruned away by active regression (Franco et al., 2013). Blood flow triggers several effects such as vessel constriction, EC survival, alignment, and migration that can all contribute to vessel regression (Meeson et al., 1996; Chen et al., 2012; Kochhan et al., 2013; Udan et al., 2013; Lenard et al., 2015; Franco et al., 2016). Interestingly, EC ability to respond to flow seems to be not fixed but dynamically adjusted according to the environment (Kwon et al., 2016). The literature shows many results on the ability of cells not only to respond to a change in flow magnitude but also to sense the direction of flow to control vessel regression. Vascular regression is driven by EC polarization and migration away from low to high flow (Chen et al., 2012). Above a critical value, flow induces axial polarization and migration of ECs against the flow direction. ECs actively migrate from regressing vessel segments to integrate into neighboring vessels (Udan et al., 2013; Franco et al., 2015). Non-canonical Wnt signaling was shown to control vascular remodeling by blocking excessive vessel regression in a flow-dependent manner (Franco et al., 2015, 2016). EC migration against the flow and vessel diameter remodeling emerge as an important mechanism that determines embryonic arterial formation. In this process, the SMAD signaling pathway, including DACH1 and endoglin (ENG), seems to play an important role (Rochon et al., 2016; Poduri et al., 2017). Smad4 deficient coronary ECs are unable to migrate against the direction of the flow and lead to an abnormal accumulation of cells within the arteries. Dach1 supports coronary artery growth through its regulation by blood flow-guided EC behavior (Chang et al., 2017). *Dach1* deletion in ECs decreases artery size with impairment of EC polarization, which could be due to inefficient directed migration against the flow. Two recent studies using mice and zebrafish mutant and *in vitro* experiments show that ENG controls flow-directed migration and EC shape required for correct vascular morphogenesis (Jin et al., 2017; Sugden et al., 2017). ENG appears to be required for proper EC sensing of both amplitude and direction of shear stress, although some aspects of EC flow-sensing were not impaired in *Eng* mutant (Bautch, 2017).

Taken together, these data show that WSS sensing, both in magnitude and direction, induces spatialized EC responses, in particular EC migration against blood flow. These responses are critical determinants of the architecture of the vascular network during development by vessel regression/stabilization and vessel diameter remodeling.

## Shear Stress Sensing: Sensors or Tensegrity?

The previous section has summarized the major consequences of WSS on the physiology of the vasculature and the experimental evidence of the influence of WSS on vascular cell behavior, what is called WSS sensing. The present section will discuss the models of WSS sensing. Authors working on WSS sensing usually produce models of it, and some of them have been presented in the previous section. Typically, the “model” of PIEZO-1 activation presented by Iring et al. (2019) is an example of a model of WSS sensing. A model of WSS sensing can be basically defined as a representation of biological processes that provide explanations of how WSS induces cellular behavioral changes, and how these changes determine the physiology of the vasculature. It goes beyond the experimental results and articulates them in a network of cause-consequence processes. In this meaning, a model provides causal explanations, namely, it explains a phenomenon by a concatenation of cause-consequence processes. A model of WSS sensing is hence an explanatory framework that gives sense to the experimental results. We propose to classify these models in two main general categories that differ in the kind of explanations they provide, the “sensor-pathway” model and the “tensegrity” one.

### The “Sensor-Pathway” Model

In WSS sensitivity of ECs, WSS can be viewed as acting like a pharmacological agent (Fung, 2010). This is obviously a metaphor, since WSS is not a molecule, but the analogy may be relevant in the meaning that WSS is sensed by the ECs and results in change in EC behavior. In the pharmacological stimulus-response coupling, the detection of the presence of the agonist is ensured by the receptor of the agonist. This receptor is hence the primary sensor of the stimulus. The ligand–receptor molecular interaction guarantees the sensitivity and the specificity of the signal detection, and the downstream pathways are responsible for the behavioral changes induced by the pharmacological stimulus. Such a model can be called the “sensor-pathway” model. However, in the case of WSS sensing, where the stimulus is not pharmacological, primary sensing cannot be ligand–receptor interaction. If the “sensor-pathway” model is relevant, the question than arises about the nature of the primary sensor of the WSS, and abundant literature exists about the possible sensors and mechanotransducers of WSS. This terminology requires some conceptual clarification. A primary sensor is a molecule or a molecular complex that is directly submitted to the WSS and is the initial trigger of the downstream pathway. A mechanotransducer is able to transduce a mechanical stimulus into a biochemical process (e.g., phosphorylation). A primary sensor can be a mechanotransducer, but a mechanotransducer is not necessarily a primary sensor.

The most frequently cited candidates as WSS sensors or mechanotransducers are primary cilia, the apical glycocalyx, ion channels such as PIEZO-1, G protein-coupled receptors, protein kinases, and caveolae. The best-studied is the EC–EC junctional complex, composed of PECAM-1, VE-cadherin, and the VEGF receptors 2 and 3. In this model, WSS induces an increase in tension across PECAM-1, mediated by an association with vimentin (Conway and Schwartz, 2015). This leads to the activation of a Src family kinase, which in response phosphorylates and activates VEGFR2, which in turn stimulates PI3K-AKT signaling pathway (Tzima et al., 2005). More recently, a study identified VEGFR3 as a novel component of this complex (Coon et al., 2015). In this model, VE-cadherin acts as an adaptor for the transmission of mechanical signal to VEGFR2 and 3. The components of this complex seem to be particularly important in the sensing of shear stress intensity and in the establishment of remodeling. Indeed, *PECAM-1*^–/–^ mice show defects in inward and outward remodeling in a partial carotid ligation model (Chen and Tzima, 2009). Recent studies have shown that VE-cadherin Y658 phosphorylation was modulated by WSS intensity (Orsenigo et al., 2012) and that this Y658 phosphorylation was crucial for flow sensing through the junctional complex (Conway et al., 2017). VEGFR3 expression was also modulated by WSS intensity, and the level of VEGFR3 was also found to participate in WSS sensing. This study showed that high expression of VEGFR3 was correlated to higher sensitivity to WSS while low expression of VEGFR3 decreased WSS sensitivity. Thus, VEGFR3 expression seemed to determine the standard value for vascular remodeling (Baeyens and Schwartz, 2016).

In addition to the requirement of the junctional complex for shear stress sensing, FAs that ensure the anchorage of ECs to the underlying basement membrane seem to be important in this process (Ando and Yamamoto, 2013). Recently, it has been shown that laminin 511, a key component of endothelial basement membrane, was essential for mouse resistance artery WSS response and inward remodeling (Di Russo et al., 2017). Cells are anchored on the basal membrane by FA via integrins, which have been showed to be a sensor of shear stress direction in ECs. For example, β1 integrins are directly sensitive to mechanical forces and are essential for EC response to unidirectional flux, via activation of Ca^2+^ signaling (Xanthis et al., 2019).

Endothelial cell primary cilia can also act as a mechanosensor, triggering calcium signaling and NO production *in vitro* (Nauli et al., 2008). Studies have shown that the presence of endothelial cilia was regulated by WSS intensity *in vivo*, and contributes to WSS-dependent vessel development. In the embryonic heart, endothelial cilia were found in low shear stress vascular regions whereas in arteries ECs were unciliated (Hierck et al., 2008). In the developing zebrafish embryo (Goetz et al., 2014) and during vascular remodeling in the mouse retina (Vion et al., 2018), primary cilia was most frequently observed in ECs exposed to low and moderate shear stress. Inducible genetic deletion of primary cilia in ECs during postnatal retina development causes premature and widespread vessel regression (Vion et al., 2018). However, since the presence of primary cilia depends on the nature of WSS, they are hence a consequence of WSS, not a primary sensor. The question remains also of how these cilia transduce into intracellular pathways the mechanical forces to which they are sensitive.

As already mentioned about WSS-induced vasoreactivity, the ion channel PIEZO-1 is a mechanotransducer and is considered in some models as the primary sensor of WSS (Iring et al., 2019). However, this model does not account for the fact that PIEZO-1 does not seem to be primarily activated by WSS but activated downstream G protein activation (dela Paz and Frangos, 2019), and that its activation requires the integrity of the cytoskeleton (Sun et al., 2001). Actually, there are two ways to conceive WSS sensing by PIEZO-1. The first one refers to PIEZO-1 as a stretch-activated channel, in which PIEZO-1 is supposed to be sensitive to the stretching of the lipid bilayer produced by the shear stress of the plasma membrane, and can be called the “force-through lipid” sensing. In this view, according to the “sensor-pathway” model, PIEZO-1 is the primary sensor. The second one refers to PIEZO-1 as sensitive to the forces exerted by the cytoskeleton, a “force through filament” sensing. Actually, there is experimental evidence for both types of force sensing but, regarding WSS, whether PIEZO-1 acts as primary sensor remains controversial (Nourse and Pathak, 2017).

From this abundant literature, some key notions emerge about mechanotransducers, in particular the importance of the junctional complex of the adherens junctions (AJs), with PECAM-1, VE-cadherin and VEGFRs, and of FAs and integrins, and the role of PIEZO-1 in WSS vasoreactivity. However, two main questions remain open. As far as the “sensor-pathway” model is relevant, the identification and the nature of the primary sensors remain problematic. Due to their locations, AJs and FAs, though sensitive to mechanical stimulation, are not directly submitted to WSS and can be hardly considered as primary sensors. Second, and most importantly, a relevant model of WSS sensing should be able to link the initial cause (WSS) to its final consequence (cell behavior) by a series of processes that retain all the spatiotemporal informative content of the stimulus (intensity, directionality, pulsatility, and linearity of WSS). In the sensor-pathway model, the cascade of cause-consequence processes from the primary sensor activation and downstream is described in terms of levels of protein expression, phosphorylation/dephosphorylation, and molecular interactions. However, these molecular processes, by themselves, do not ensure the conservation of the spatiotemporal characteristics of WSS. As stated by Baeyens, “a coherent model of flow sensing is lacking” (Baeyens et al., 2016). This questions the relevance of the classical “primary sensor-downstream pathways” model, inherited from the pharmacological conception of ligand-receptor sensor, for the investigation and the understanding of WSS sensing.

### The “Tensegrity” Model

In the usual conception on which the “sensor-pathway” model is grounded, a cell is most of all viewed as a viscous protoplasm limited and contained by an elastic membrane. An alternative view is the “tensegrity” model of mechanosensing (Paszkowiak and Dardik, 2003). The concept of tensegrity, or tensional integrity, coined by Richard Busckminster Fuller, is initially an architectural principle (Fuller and Applewhite, 1982). The mechanical stability of the structure built according to this principle is ensured by a net of continuous tension exerted on the components of the structure that are either in tension or in compression. A camping tent is an example of tensegrity. The shape of the tent is the consequence of the equilibrium of the tensile forces to which the different components of the tent, e.g., tent canvass, poles, ropes and pegs, are submitted, each one being balanced by an equal one opposite in direction. Any local change in tension alters this equilibrium and hence has global consequences on the shape of the structure that rearranges until the tensile forces reach a new equilibrium. But, as far as the tensional net is maintained, the structural integrity is retained despite the change in its shape. A structure is a tensegrity when it is in a state of baseline isometric tension, or tensional prestress, which avoids any slack in the tensional structure. This makes it both resilient and immediately responsive to internal and external mechanical stresses.

The concept of tensegrity has been applied to several biological processes at different levels of organization, including cellular mechanosensing (Ingber, 1997, 2008). In the model developed by Ingber (2018), the cell is viewed as shaped by the cytoskeleton whose architecture is ensured by tensional prestress. The three main components of the cytoskeleton are (i) the microfilaments, containing actin and myosin), (ii) the intermediate filaments, basically composed of vimentin, keratin, and desmin, and (iii) the microtubules, hollow polymers of tubulin. All of them contribute to this tensional prestress. The contractile microfilaments generate the active tension to which intermediate filaments and microtubules are submitted. The cytoskeleton is linked to FAs, which are transmembrane macromolecular structures containing talin, vinculin, α-actinin, paxillin, and integrin. Integrins bind with the extracellular matrix, so that FAs are molecular bridges between the cytoskeleton and the extracellular matrix to which it is anchored via integrins. By experimental tuning of the mechanical forces exerted on the cell, it has been evidenced that the cytoskeleton mechanically associated with the extracellular matrix behaves as a tensegrity structure (Kumar et al., 2006; Ingber, 2018). When the cell is submitted to mechanical deformation, the forces exerted on the tensional network are modified, resulting in changes in the tractional forces on FAs and integrins receptors. These changes in the balance of forces activate several biochemical processes, in particular the activation of small GTPases Rho that modulate F-actin and hence actomyosin-dependent tension generation by contractile microfilaments (Ohashi et al., 2017). A recent minimal theoretical model showed that feedback between mechanical tension and Rho GTPase activity can account for different kinds of spatially organized cell behaviors, such as individual cell relaxation/contraction state, and the propagation of contraction waves in a 2D sheet of interconnected cells (Zmurchok et al., 2018).

While several studies have used tensegrity (whether they use the name or not) to provide an explanatory framework of mechanosensitivity, few studies using tensegrity have been specifically dedicated to WSS mechanosensing in ECs (Lim et al., 2015). Among them, a multicomponent, multicell model of the mechanical effects of FSS on ECs has been recently published (Dabagh et al., 2014). Using the finite-element method, the authors have built a computational model that predicts the deformation of ECs and subcellular organelles, and the stress to which the cytoskeletal stress fibers, the cell–cell AJs, and the cell-matrix FA are submitted when exposed to 1–2 Pa WSS, a value in the physiological range. The model predicts deformation of the cell and the nucleus, change in junctional angles, and an increase in AJ and FA stress. Indeed, the stress at FA increases up to 480 Pa and that at AJ up to 700–1,200 Pa. This corresponds to a 250–600 fold amplification of the intensity of the WSS at FAs, and a 600-fold amplification at AJs. The stress values predicted by the model are also in the range of order (kPa) of the stress values experimentally measured at FAs (Balaban et al., 2001).

According to the model, the architecture of the cell is hence responsible for the transmission and the amplification to the AJ and the FA of the FSS to which the luminal surface of the EC is submitted. In epithelial cells, some experimental results also suggest that changes in cytoskeleton tension are the initial events required for the response to FSS. In cultured MDCK cells, using optical force sensor for α-actinin, and fluorescent E-cadherin, it has been shown that shear stress-induced remodeling of AJs is driven by cytoskeletal forces (Verma et al., 2017). In ECS, as we have seen previously, several experiments have evidenced the key role of the cytoskeleton integrity for WSS sensing.

There are hence several theoretical and experimental results that strongly suggest that tensegrity is responsible for WSS sensing in ECs. In this view, “sensing” is primarily a change in the tensional equilibrium of forces operating in the cytoskeleton, cell-substrate, and cell–cell adhesions, due to the deformation induced by FSS at the lumen surface of the EC. The “primary” sensor, able to transmit and amplify the mechanical stimulus, is not an individualized molecular component of the cell directly submitted to FSS, but the cell architecture as a whole. However, WSS mechanosensing is not limited to mechanical processes. Cellular active processes such as phosphorylation, gene expression, etc., are involved in the cell response to FSS. These processes are physiological ones that are not energetically spontaneous and are distinct from the passive ones, occurring without energy consumption. This makes a critical difference between a passive tensegrity structure, in which the initial external force exerted locally induces a global change in shape, and a biotensegrity, in which active processes are involved. In the biotensegrity approach, these physiological processes are generated by the structural changes in the whole-cell shape induced by FSS, and are the consequence of mechanochemical transducers. They trigger several biochemical processes (e.g., cadherin expression, Rho activation, and microtubule polymerization) in response to the WSS-induced architectural changes. Second, if these biochemical processes modify the tensegrity equilibrium of the cell, which is spatially oriented, this may explain the conservation of the spatiotemporal characteristics in the WSS stimulus-response coupling.

The main difference between the “sensor-pathway” and the “biotensegrity” models does not reside in the molecular components involved in FSS mechanosensing, but in the nature of the explanations of WSS-response coupling they provide. In the “sensor-pathway” concept, the investigation of the mechanisms responsible for WSS-sensing focuses on the identification of molecular interactions and biochemical processes, which indeed occur during mechanosensing, but pay little attention to how these processes are responsible for the spatially oriented behavioral responses of the cells. The biotensegrity concept focuses on the causal continuity of the stimulus-response coupling, explained by the tensional equilibrium of the cell architecture. An important point is that, in the biotensegrity concept, this tensional equilibrium is not just passive adjustment to external mechanical constraints but also includes active internal adjustment due to the activation of biochemical processes. Hence, the biochemical “pathways” are embedded in a network of tensional forces, in the meaning that they are triggered by mechanical changes (via mechanochemical transducers), and modify the tensional equilibrium of the cellular architecture. They hence contribute to the cell response to WSS because they interplay with the structural organization of the cells. The biotensegrity model, which integrates the WSS-dependent biochemical pathways as active internal components of the tensegrity, is not just a model of mechanosensing (how the cell senses WSS) but also a model of mechanosensitivity (how the cell responds to WSS).

## Uniform Shear Stress and Set Point Theory

### Theoretical Formulation

As we have seen, ECs are able to sense the spatiotemporal characteristics of WSS and therefore contribute to determine the morphofunctional properties of the vascular network by at least three kinds of mechanisms, namely vessel regression and stabilization, long-term modulation of vessel diameter by inward and outward remodeling, and short-term vasoreactivity. Moreover, the consequence of these WSS-dependent processes is a retroactive limitation of WSS variation. These observations have lead Baeyens and coworkers to apply the “set point” theory of regulation to endothelial WSS sensitivity (Baeyens et al., 2015, 2016; Baeyens and Schwartz, 2016). Their model was proposed for WSS-induced vessel diameter remodeling, but the concept can also be applied to others vascular properties.

The “set point” theory, or “target point” theory, is a model of regulation of a biological process in which a biological variable remains in a determined range of values despite the environmental changes that modify the initial value of the variable. Applied to WSS sensing, it means that there exist mechanisms that ensure that the WSS characteristics remain in a small range of values despite changes in the conditions that determine WSS in the vessel (i.e., changes in flow rate or velocity, changes in blood viscosity). Expressed in bioengineering terms, such a phenomenon of regulation can be formulated as follows (Fung, 2010):

(a) A variable *x*, or a relationship among a set of variables (*x*, *y*, …) that describes the phenomenon has been identified.
(b) There exists a standard value of x or a standard relationship among (*x*, *y*, …) that is associated with a stable or optimal living condition.
(c) There exists a sensor that can detect any deviation or error of the variable x from the standard value, or of the relationship among (*x*, *y*, …) from the standard one. The error is monitored all the time.
(d) A mechanism to minimize error exists.
(e) The dynamics of error minimization is biologically satisfactory.

According to this definition, once the variable (WSS) and its standard value have been identified, some key conditions are required to characterize a regulation process. The first one is how can be objectivized the “optimal” living conditions and the adequacy of the biological response (the fact that it is “satisfactory”), notions to which refer to items (a) and (b) of the definition. The second one is the identification of the mechanisms responsible for deviation minimization, which refers to item (c) of the definition. A possible way to define the biological optimization of a vascular network is to consider the minimal energy cost of blood flow. This notion has been formulated since the first half of the 20th century. In a branching network through which flows a fluid, minimum energy expenditure is achieved if the resistance to blood flow remains constant from proximal to distal generations (Fung, 2010). In this minimum cost model, for each vascular bifurcation, the relationship between the radius of the parent segment of generation *n* (*r*_*n*_) and the radius of the two child segments (*r*_(_*_*n*_*_+1)_*_*a*_*) and (*r*_(_*_*n*_*_+1)_*_*b*_*) is:

(10) $$r_{n}^{3}=r_{\left(n+1\right)\,a}^{3}+r_{\left(n+1\right)\,b}^{3}$$

When applied to WSS, the minimum cost model shows that this condition is achieved when the WSS is equal in all of the segments (Kamiya et al., 1984), due to the fact that WSS is inversely proportional to *r*^3^ (Eq. 7). So, if the WSS value is similar in all of the segments of the vascular tree, this ensures the energetic optimality of the vascular architecture. This has led to the formulation of the “uniform shear stress” principle, responsible for “optimal design,” as formulated by Kamiya et al. (1984). According to this principle, maintenance of WSS value in each part of the vascular tree by local adaptive response to WSS change ensures the energetic optimality of the entire arterial tree. In principle, this can be applied to the three above-mentioned mechanisms, namely, vascular morphogenesis, long-term vascular remodeling, and short-term vasoreactivity. In the case of vessel regression and stabilization, an exuberant process of vessel sprouting and random connection, followed by vessel regression below a threshold WSS value, associated with vessel inward or outward remodeling of the remaining vessels until the set point value is obtained, will produce an organized network optimally designed. Uniform shear stress in vascular morphogenesis can hence be viewed as a constructal process that tends to shape vascular networks following thermodynamic constructal principles (Roux and Marhl, 2017). Adult vascular remodeling can also be analyzed in term of uniform shear stress. Indeed, as argued by Bayens et al., inward and outward remodeling are feedback processes ensuring the maintenance of constant WSS. WSS-induced vasoreactivity can be also analyzed as a negative control of WSS. Since this negative feedback maintains the energetic optimality of the vasculature, it can be said to be adaptive.

**FIGURE 2:**
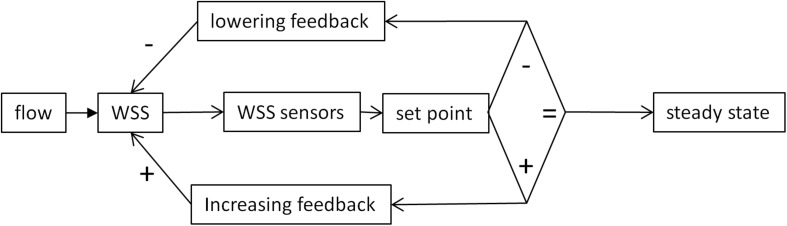
Schematic representation classical set point theory according to the concepts of control theory. The WSS value is sensed by mechanosensors and compared with a reference value. If the two values are similar, the system remains in a steady state. If not, positive or negative variations from the set point activate feedback mechanisms that, respectively, lower or increase WSS value. The diamond represents the decisional step of the regulatory loop.

This adaptive response requires a mechanism responsible for deviation minimization. Actually, since WSS sensing is involved in different processes (vasoreactivity, morphogenesis, and remodeling), acting on different time scale, different mechanisms operate in this feedback. However, these different mechanisms can be analyzed with the common concept of homeostasis. Formulated in accordance with the control theory applied to physiological homeostasis (Carpenter, 2004), the principle of such mechanisms can be represented as an algorithmic process monitoring the WSS value by comparison with a set point value (Figure 2).

### Uniform Shear Stress and Physiological WSS Sensitivity

In order to examine the relevance of the uniform shear stress principle, and the adequacy of the SPT as a suitable concept for analyzing the WSS sensitivity mechanisms, quantitative experimental data need to be compared with the theoretical requirements of the theory. The uniform shear stress principle has been primarily formulated for WSS-induced vessel remodeling, and several studies have shown that, after remodeling, the WSS remains unchanged (Kamiya and Togawa, 1980; Zarins et al., 1987). For example, in monkey iliac artery, after outward remodeling following arterovenous fistula, WSS, initially 16 dyn cm^–2^, was 15 dyn cm^–2^ (Zarins et al., 1987). Long-term remodeling adaptation seems hence to follow the uniform shear stress principle. As stated by Baeyens and Schwartz (2016), this can be explained by the balance between quiescent (no remodeling) and active (inward or outward remodeling) states. Quiescence can hence be associated to different diameters.

For short-term vasoactive response to WSS, such a process is not possible. It is certainly possible to define in theory a quiescent state, corresponding to the absence of vasoactive stimulus from ECs to the smooth muscle layer, and a corresponding “set point” value. But the value close to which WSS sensitivity tends to maintain WSS cannot correspond to the quiescent state. Indeed, for the system to be able to react both to WSS increase or decrease, the “set point” should be in the range of WSS sensitivity, and hence corresponds to a vasoactive state. Moreover, WSS-induced vasodilatation requires change in WSS. If the response was able to maintain uniform WSS, the consequence would be the annihilation of the WSS change, and subsequent ending of the vasoactive stimulus, and return to the initial vessel diameter. In contrast with remodeling, in WSS-induced vasoreactivity, stable WSS value is not quiescence, but a dynamic equilibrium between two phenomena, the physical relationship between vessel radius and WSS described by Eq. 6 and the physiological WSS sensitivity that links WSS and the vessel radius. Acting together, these two phenomena constitute a feedback loop. We have analyzed several quantitative data of WSS-induced vasoreactivity *in vivo* already published in the literature, in which the authors have experimentally manipulated the blood flow rate and measured the consequence of flow rate change on vessel diameter and/or WSS. Data, taken from several vascular beds, human CCA, human brachial artery (BA), and rat cremaster arterioles (CrA), are summarized in Table 3. Since the authors had not always calculated the flow rate, the vessel diameter, and the WSS or WSR, we have applied Eq. 6 to calculate the missing data. For the calculation of WSS from WSR, blood viscosity was set at 0.035 Poise for large arteries, and 0.02 for arterioles, in order to take into account the decrease in apparent viscosity in small vessels, in accordance with experimental measurements (Kamiya et al., 1984). In large arteries, WSR ranges from 188 to 275 s^–1^, and WSS from 7 to 20 dyn cm^–2^, values that are in the physiological range. In arterioles, WSR is around 10-fold more, and WSS around fivefold more. These experimental data are in accordance with the proportionality between WSS and pressure differential described in Eq. 8. Indeed, terminal arterial segments are the site of the largest decrease in blood pressure, and hence of highest WSS. This confirms the fact that WSS is not uniform all along the arterial tree.

For a given kind of segment, change both in WSS and diameter values induced by increase in blood flow rate confirms that there exists WSS-induced vasoreactivity (since diameter changes) but not uniform WSS (since WSS increases). We can hence define a WSS sensitivity coefficient, *S*_*WSS*_, as the ratio of vessel radius difference on WSS difference between initial and final blood flow rate. In the absence of WSS sensitivity, *S*_*WSS*_ = 0, while, for uniform shear stress, *S*_*WSS*_→∞.

From the experimental data given in Table 3 and Eq. 6, it is possible to build a graphical representation of the function *r* = *f*(*WSS*), *r* being the radius of the vessel. Indeed, from Eq. 6:

(11) $$r=\sqrt[{3}]{4\,\eta \times \frac{Q}{\pi \,{\mathrm{WSS}}^{3}}}$$

**FIGURE 3:**
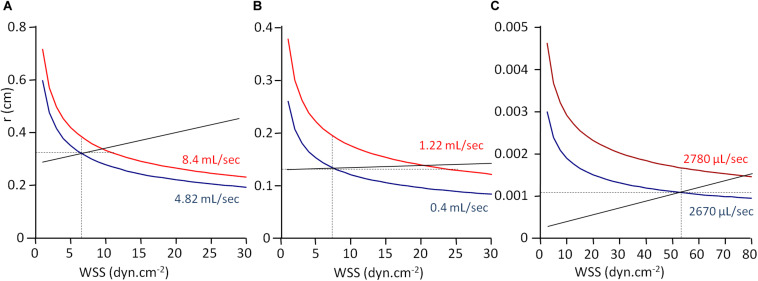
WSS-induced vasoreactivity. Graphical representation of WSS and vessel radius from Table 3 data. *r*, vessel radius; WSS, wall shear stress. The curves are the graphical representation of the function *r* = *f*(WSS) calculated according to Eq. 11. Values for blood viscosity (η), blood flow (*Q*), *r*, and WSS are the experimental ones given in Table 3, and the curve is built by varying WSS value and calculating *r* from the equation. Panels **(A)**, **(B)**, and **(C)** are the curves built from the data obtained in human common coronary artery **(A)**, human brachial artery **(B)**, and rat cremaster muscle arterioles **(C)**. Full black lines represent WSS sensitivity, the slope of the line being the sensitivity coefficient *S*_*WSS*_. Intercepts of the line with the curves are the observed radius and WSS values. Horizontal and vertical dot lines correspond, respectively, to the absence of WSS sensitivity, and uniform WSS.

For a given blood flow rate *Q* and a given blood viscosity η, the vessel radius in inversely proportional to the cubic square of WSS. Since *Q* and η are known, the function *r* = *f*(*WSS*) can be built, adjusted to the couple of experimental values for r and WSS. For each arterial segment (CCA, BA, and CrA), *r* = *f*(*WSS*) is given in Figure 3, for both initial and final conditions. Vascular adjustment in response to increased blood flow rate corresponds to the shift from the initial curve to the final one. In the absence of vasoactive response, the vessel diameter remains constant, and this shift is horizontal. Hence, the horizontal intercept from the initial WSS and radius with the final curve gives the final WSS in the absence of WSS sensitivity. Under the hypothesis of uniform WSS, WSS sensitivity ensures the conservation of WSS. In this case, the shift from the initial to the final curve is vertical. Hence, the vertical intercept from the initial WSS and radius with the final curve gives the final radius in the case of maximal WSS sensitivity (strict maintenance of WSS). Actually, the real situation is between these two opposite hypotheses. The straight line between the initial and the final WSS and radius values represents the real WSS sensitivity, and the slope of this line is *S*_*WSS*_. As can be seen in Figure 3 and Table 3, *S*_*WSS*_ is low, so the effect of WSS sensitivity of vessel radius is modest. This does not mean that it is physiologically unimportant in terms of vascular resistance, because the resistance is inversely proportional to the radius at the power 4. In the absence of WSS sensitivity, the resistance would be greater.

About vessel regression during vascular morphogenesis, the situation appears more complex. Indeed, the first stages of vascular development occur in the absence of blood flow. The initial vascular network should be hence flow-compatible, but is not flow-directed. When flow occurs, WSS sensitivity can take place and contribute to vascular remodeling.

In the model proposed by Franco et al. (2016), with no or reduced flow, ECs do not rearrange according to flow, whereas, above a threshold value, WSS sensitivity takes place. The axial polarity vector (the nucleus to Golgi apparatus vector) of ECs orients against the flow, and ECs migrate from low to high flow zones (Franco et al., 2016). Computational modeling of blood flow and WSS in newborn mouse retinal vascular plexus showed very high WSS values, up to 10 Pa (100 dyn cm^–2^) close to the optical nerve, at the origin of the plexus, with a more or less linear drop to a WSS value below 2 Pa (20 dyn cm^–2^) at 1 mm distance from the origin of the plexus (Franco et al., 2016). Cell density decreases with WSS, but not linearly. There seems to be a plateau in vascular density close to 3 Pa (30 dyn cm^–2^), while EC polarization against the flow (axial polarity vector anti-parallel to flow) is linearly correlated with WSS value. This is compatible with the existence of a WSS value in reference to which vessels stabilize, though further studies are needed to experimentally support the existence of a set point value below which vessels regress and above which they stabilize.

### Set Point Theory and Dynamic Equilibrium

Taken together, these experimental data support the existence of regulatory WSS-sensitive mechanisms that tend to stabilize WSS by short-term and long-term vessel diameter adjustments, and suggest the existence of a critical WSS value for vessel regression/stabilization during vascular morphogenesis. The uniform shear stress theory, as a physical principle, and the SPT, as a physiological one, constitute a relevant conceptual framework to analyze the different kinds of WSS sensitivity. However, the uniform shear stress principle should be viewed as an idealization of the vascular network that is not strictly verified experimentally. WSS value is not uniform all along the vascular bed. Moreover, in some cases, as we have seen for WSS-induced vasoreactivity, the mechanisms for WSS regulation are incompatible with strict maintenance of WSS. This does not invalidate the idea that local sensing of WSS contributes to the overall architecture and efficiency of the vascular network, but raises the question of how the reference WSS value is adjusted locally.

**FIGURE 4:**
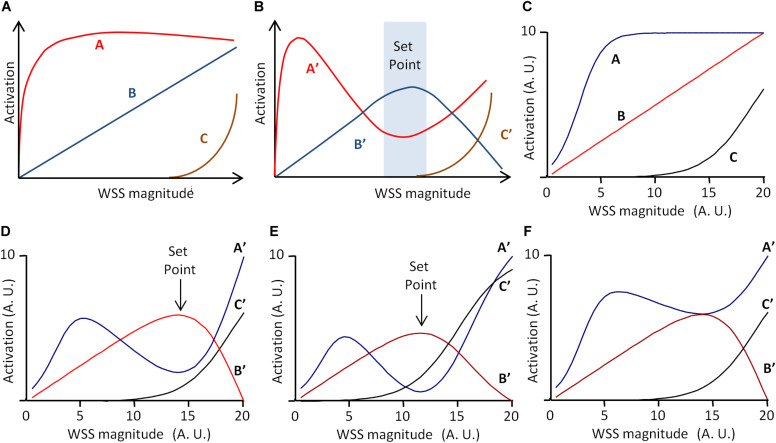
Scheme of the set point model for vascular remodeling. **(A,B)** Scheme of the dynamic model proposed by Baeyens and Schwartz (2016), redrawn from the original publication. A, B, and C are three WSS-dependent intercellular pathways determining vessel remodeling and quiescence. **(A)** Independent activation profile of A, B, and C. **(B)** Activation profile of A, B, and C outputs (A′, B′, and C′, respectively), when C inhibits B, and B inhibits C. If A′ determines inward remodeling, B′ quiescence, and A′C′ outward remodeling, the model predicts a “set point” zone of WSS for which B′ is greater than A′ and C′, corresponding to the quiescent state. **(C)** Graphical representation of the mathematical formulation of the model (Eqs 12–17) with *A*_*m*_ = 10, *C*_*m*_ = 10, α = 3, β = 0.5, γ = 19, *ha* = 0.4, and *hc* = 0.2. **(D)** Model prediction for A′, B′, and C′ for *I_*c*_* = 100% and *I_*b*_* = 80%. **(E)** Model prediction for A′, B′, and C′ for γ = 15 instead of 19. Values are expressed in arbitrary units (A.U.). **(F)** Model prediction for A′, B′, and C′ for *I_*b*_* = 40% instead of 80%. Vertical arrows indicate the set point value, defined as maximal B′ value above A′ and C′ values. Values are expressed in arbitrary units (A.U.).

Another important issue is how we conceive and represent the mechanisms that minimize deviation from the set point value. As exposed above, in the classical representation of regulatory mechanisms, grounded on the theory of information, regulatory processes are viewed as a flowchart of instructional steps. As highlighted by Baeyens and Schwartz (2016), such an algorithmic view of set point maintenance is unlikely to account for the real processes occurring during vascular remodeling. Instead, they have proposed a model including three pathways, denoted A, B, and C, of different WSS sensitivities and effects on vessel remodeling (Baeyens and Schwartz, 2016). The model presented by the authors is given in Figure 4A. A reaches its maximum for low WSS, C begins to increase for high WSS, and B increases linearly with WSS. Additionally, A, B, and C interfere as follows: B inhibits A and C inhibits B. Integration of these interactions produces the following patterns for A, B, and C outputs, denoted A′, B′, and C′, given in Figure 4B. A′ is maximal for low WSS, and then decreases while B′ increases. B′ is maximal for middle WSS value and C′ increases with high WSS values. Considering that high B′ induces quiescence, high A′ inward remodeling, and high A′C′ outward remodeling, the quiescent state occurs for a specific WSS value determined by a dynamic equilibrium between these three pathways. This value can be said the “set point” value (Baeyens and Schwartz, 2016).

In their article, the set point model published by Baeyens and Schwartz (2016) was presented graphically, but it can be described mathematically. This allows more precise prediction of the pathway outputs A′, B′, and C′, and the existence of a set point value for WSS. We hence propose the following mathematical description, in which A and B are described by two sigmoidal equations and B by a linear one.

(12) $$A=\frac{A_{m}}{1+10^{{\left(\alpha +\mathrm{WSS}\right)}^{\mathrm{ha}}}}$$

(13) B=β×WSS

(14) $$C=\frac{C_{m}}{1+10^{{\left(\gamma +\mathrm{WSS}\right)}^{\mathrm{hc}}}}$$

These equations are purely descriptive, and the parameters *A*_*m*_, *C*_*m*_, α, β, γ, *ha*, and *hc* have been chosen so that, for WSS varying from 0 to 20 (arbitrary units), the profile of the equations corresponds to the profile of the pathways in the original publication (Figure 4C). The inhibitions of B by C and of A by B are described by two inhibitory coefficients *I*_*C*_ and *I*_*B*_, respectively. A, B, and C outputs, denoted A′, B′, and C′, are then described as follows:

(15) $$C^{\prime }=C$$

(16) $$B^{\prime }=B\times \left(1-I_{C}\times \left(\frac{C^{\prime }}{C_{\max}^{\prime }}\right)\right)$$

(17) $$A^{\prime }=A\times \left(1-I_{b}\times \left(\frac{B^{\prime }}{B_{\max}^{\prime }}\right)\right)$$

$C_{m\,a\,x}^{\prime }$ and $B_{m\,a\,x}^{\prime }$ are the maximal values of C′ and B′ for WSS varying from 0 to 20. Figure 4D shows the resulting profiles of A′, B′, and C′ for *I*_*C*_ = 100% and *I*_*B*_ = 80%. These profiles are similar to that presented by Baeyens and Schwartz (2016). The curves show indeed a “set point” value, corresponding to the WSS value for which B′ is maximal and superior to A′ and C′. This set point does not exist for each pathway taken individually in the absence of inhibitory interactions, but is the consequence of the dynamic equilibrium between A, B, and C and their inhibitory interactions. As illustrated in Figure 4E, change in the activation profile of one pathway results in change in the set point value. This may explain how similar pathways may lead to local differences in set point values through local changes in their activation profile. Additionally, if we define the robustness of the system by the amplitude of the difference between B′ (quiescence) and A′ and C′ (remodeling), the model explains how the robustness of the quiescent state can vary locally. The limit case is illustrated in Figure 4F. If *I*_*B*_ = 40%, half of its initial value, then A′ is always superior or equal to B′. This shows that change in these interactions can lead to the loss of the quiescent state, and hence loss of the physiological equilibrium of the vessel.

**FIGURE 5:**
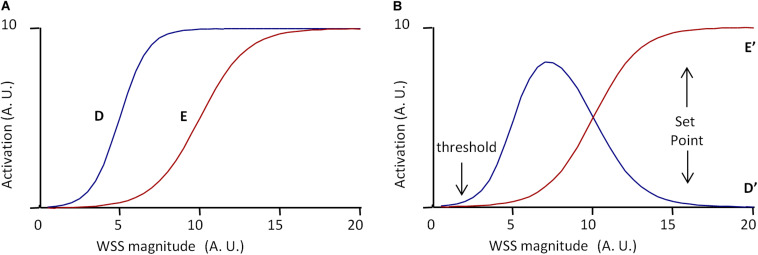
Scheme of the set point model for vessel stabilization. The model is based on two pathways, D and E, defined by Eqs 18–21, E inhibiting D. **(A)** Independent activation profiles of D and E with *D*_*m*_ = 10, *E*_*m*_ = 10, δ= 5, ϵ= 10, *hd* = 0.5, and *hc* = 0.3. **(B)** Predicted activation profiles of D and E outputs (D′ and E′, respectively), for *I_*e*_* = 100%. Values are expressed in arbitrary units (A.U.). The model predicts a minimal WSS threshold for WSS sensitivity. If D determines cell migration and E cell polarization, and if vessel stabilization is defined by maximal EC polarization and the end of EC migration, the model predicts a set point value for vessel stabilization, cell migration being maximal before the set point value is obtained.

Such a model can be adapted to vessel regression/stabilization, considering that vessel stabilization corresponds to a quiescent state for EC migration. One possible model is presented in Figure 5. In this case, only two pathways denoted D and E are considered, E inhibiting D. D and E are described by two sigmoidal equations.

(18) $$D=\frac{D_{m}}{1+10^{{\left(\delta +W\,S\,S\right)}^{h\,d}}}$$

(19) $$E=\frac{E_{m}}{1+10^{{\left(\varepsilon +W\,S\,S\right)}^{h\,e}}}$$

The parameters *D*_*m*_, *E*_*m*_, δ, *hd*, and *he* have been chosen to obtain the desired profiles presented in Figure 5A. D and E outputs, denoted D′ and E′, resulting from the inhibition of D by E, are expressed as follows:

(20) $$E^{\prime }=E$$

(21) $$D^{\prime }=D\times \left(1-I_{E}\times \left(\frac{E^{\prime }}{E_{m\,a\,x}^{\prime }}\right)\right)$$

*I*_*E*_ is the inhibitory coefficient and $E_{m\,a\,x}^{\prime }$ are the maximal value of E′ for WSS varying from 0 to 20. E′ and D′ profiles for *I*_*E*_ = 100% are given in Figure 5B. According to E′ and D′ profiles, there exists a threshold value for WSS sensing. Considering that D′ determines cell migration against blood flow, the model predicts that cells migrate from low WSS regions to higher WSS regions, until they reach a region with a WSS value corresponding to the complete inhibition of migration. If E′ induces cell polarization, the model also predicts that cells polarization increases with WSS until it reaches the same value. This WSS value can be said the “set point” value, with the same semantic limitations previously notified. In these set point models of vessel remodeling and vessel regression/stabilization, the WSS “set point” value is the consequence of a dynamic equilibrium, not the determinant of an algorithmic process. It is also locally determined, being the consequence of local interactions between several pathways.

**FIGURE 6:**
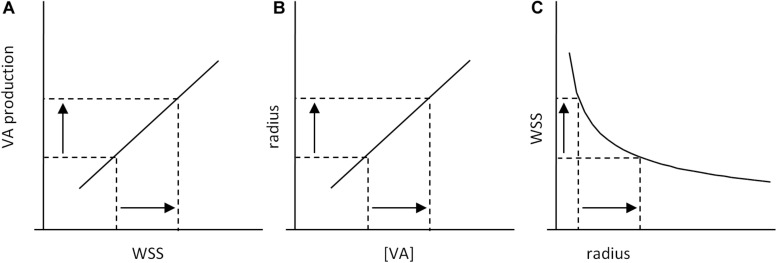
Scheme of WSS-induced vasoreactivity regulation. **(A)** WSS-induced vasorelaxant agents (VA) production by ECs. An increase in WSS induces an increase in VA production, which leads to an increase in VA concentration (VA) in the smooth muscle cells (SMC) of the vessel wall. **(B)** VA-induced vasorelaxation of SMCs induces an increase in vessel radius. **(C)** Because of the physical relationship between vessel radius and WSS, increase in radius induces a decrease in WSS. Taken together, these processes constitute a causal loop that limits WSS variations: the consequence of an initial increase in WSS is WSS decrease, and reciprocally.

Clearly, the algorithmic representation is also inadequate for WSS-induced sensitivity. Schematically, the consequence of EC WSS sensing is an enhanced production of vasorelaxant agents (VA) such as NO that induce smooth muscle cell relaxation and subsequent increase in vessel diameter. This can be ensured by a positive (e.g., linear) relationship between WSS and VA production, which induces a proportional increase in vessel diameter, and subsequent drop in WSS (Figure 6). This does not pretend to describe the precise mechanisms of WSS-induced vasoreactivity, but just to illustrate its general principles.

This representation is a simplified but relevant one of the cellular processes identified as responsible for WSS-induced vasoreactivity. These processes constitute a feedback loop that limit WSS fluctuation, but there is no set value for WSS that determines VA production. The range of WSS sensitivity has boundary values, but, within these limits, there is no threshold value for VA production that corresponds to the “normal” steady state WSS. Hence, within the sensitivity range, WSS equilibrium is ensured by threshold-free mechanisms. The “set point” value is a consequence of the existence of the processes involved in WSS sensing, but not a causal element of any of these processes. Similarly, in the developmental model of flow-dependent vascular remodeling proposed by Franco et al. (2016), we should be aware that the threshold value present in the model does not correspond to a set point value for WSS, but to the lower limit of WSS sensitivity. The representation of WSS regulation as a series of instructional steps that include measurement of WSS and comparison with a reference value does not correspond to the real operating processes. The classical terminology of “standard value,” “error,” and “error monitoring” is purely metaphoric and hence misleading. The formulation of “set point” is also ambiguous. When used, it should be interpreted as a steady state level of WSS resulting from the dynamic equilibrium of mutually interacting processes, what can be called the “dynamic set point” theory.

## Conclusion

Wall shear stress sensing by ECs is a complex process that requires the ability of the cells to integrate the spatiotemporal characteristics of the WSS and behave in a differentiate manner to its different patterns. The concept of biotensegrity seems a relevant and fruitful conceptual framework to provide causal links of the stimulus–response coupling. In such a view, the notion of biochemical pathways is embedded in a more general organizational view governed by tensional constraints. The notion of primary sensors is not really relevant, since the whole cell architecture is the sensor, and a predominant explanatory value is attributed to mechanotransducers as a key element of active modification of the tensional equilibrium on the cell.

The existence of vascular WSS sensing is a general property of vascular systems in Mammals and other animal taxons (LaBarbera, 1990; Kochhan et al., 2013). This means that, though involved in several pathologies, it is first of all a physiological property that contributes to the adequate development and functioning of the vascular system. Involvement of WSS sensing in diseases is the exception, not the rule. Hence, understanding how it can be associated, in some cases, with vascular dysfunction requires first to understand how it contributes to the normal vascular function. Under some restrictions and precisions, both conceptual and terminological, the uniform shear stress principle and the SPT provide a relevant framework to interpret how WSS-induced EC behaviors contribute to the overall organization of the vascular network. Indeed, WSS variation is limited by active processes that regulate WSS value. The set point models for vascular remodeling, vessel stabilization, and vascular reactivity presented in this article are very simplified ones. Their purpose is not to give a realistic description of the precise mechanisms responsible for WSS sensing. It is to demonstrate that interplays between WSS-induced physiological processes can generate a set point toward which the vessels tend to stabilize. The concept of WSS set point value seems thus relevant, as far as it is interpreted as the consequence of a dynamic equilibrium between physiological processes and physical constraints, and not as a reference value in an algorithmic monitoring of WSS. In this view, the “set point” terminology is useful for designating the value to which the dynamic processes tend to equilibrate, but its meaning is more metaphorical than real. The expression of “threshold value” is ambiguous, since it may be used to name two different things that should not be confounded, the WSS set point value, and the boundary values of WSS sensitivity range. Actually, in the range of sensitivity, regulatory processes that contribute to the “set point” value can be threshold-free.

The uniform shear stress principle is a relevant concept, since it is broadly verified in practice. Coupled with the dynamic SPT, and applied to both vascular morphogenesis and vessel diameter adaptation, it explains how local processes of WSS regulation can produce overall optimized design (defined in term of energy expenditure) of the vessel network. The way WSS can shape the vascular architecture is typically a self-organized process, namely, a process in which the overall organization is determined by local behavioral rules without centralized control (Seeley, 2002). The dynamic SPT also provides a possible explanation for local variations of WSS set point values. It also predicts local variations in the robustness of the quiescent state. This may explain why some vascular zones are more susceptible than others to shift from a physiological quiescent state to a pathological permanent inflammatory one. Shift from normal to pathological vessels may also be explained by the loss of the existence of the quiescent state. It can also be due to the inability of WSS-induced vessel modification to restore the initial set point WSS value.

Though the dynamic SPT provides a theoretical possible explanation for local variations of the set point WSS value, it does not fully explain the local variations of WSS normal value. If these variations are due to local differences in WSS-sensitive pathway interactions and activation profile, the question remains of the cause of such variations. It can be hypothesized that other local processes (e.g., O_2_ sensing) interplay with WSS sensing. This requires further investigation and implementation of the dynamic SPT.

Very localized variations in WSS normal values, and hence in normal WSS set point, can occur in a given vessel segment, as it happens, for example, in the aortic cross, with zones of laminar blood flow and high WSS, and others with disturbed flow and low WSS. As seen previously, models based on 3D imaging of the vascular architecture and computational fluid dynamics can predict the spatial distribution of WSS values and their temporal variations due to blood flow pulsatility (Ong et al., 2020). The principle of dynamic set point WSS locally determined remains conceptually valid. If local variations of WSS values are coupled with local variations of WSS set point, the overall stability of the vessel is maintained. Also, local cell–cell interactions in the endothelial layer can contribute to coordinate the local response of the endothelium to WSS. However, at that scale, the validity of the principle of uniform shear stress is questioned. So, though valid in principle, application of the dynamic SPT to localized variations in WSS remains highly speculative. Its theoretical formulation would require coupled computational models of fluid dynamics with modeling of WSS-sensitive cell behavior. The development of such models would be helpful to understand how a vessel segment can shift from its physiological state to a pathological one.

In summary, the concept of biotensegrity provides a relevant explanatory framework for WSS sensing, and the dynamic SPT, coupled with the principle of uniform shear stress, a relevant one to understand how local WSS sensing can lead to the global optimization of the vascular architecture. Both concepts are dynamic ones. The behavior of the cells and the architecture of the vasculature are viewed as dynamic equilibrium of tensional forces and pathway outputs. These concepts can be formulated in a mathematical way. However, realistic models of such processes remain to be developed.

## Author Contributions

ER contributed to the general conception of the manuscript, presentation of the theoretical principles of WSS, shear stress and vasoreactivity, critical analysis of tensegrity, theoretical formulation and critical analysis of uniform stress principle and set-point theory, calculation of WSS sensitivity from bibliographical data, and overall writing of the manuscript. PB contributed to the review on WSS sensitivity in angiogenesis and vascular remodeling. PD contributed to the general conception of the manuscript, review on WSS sensitivity in angiogenesis vascular remodeling in relation with set-point theory, and overall writing of the manuscript. TC contributed to the general conception of the manuscript and overall writing of the manuscript.

## Conflict of Interest

The authors declare that the research was conducted in the absence of any commercial or financial relationships that could be construed as a potential conflict of interest.
